# Do overweight/obesity and low levels of leisure-time vigorous physical activity moderate the effect of occupational physical activity on self-rated health of construction workers?

**DOI:** 10.1007/s00420-021-01771-2

**Published:** 2021-10-07

**Authors:** M. Van den Berge, S. H. Van Oostrom, H. F. Van der Molen, S. J. W. Robroek, C. T. J. Hulshof, A. J. Van der Beek, K. I. Proper

**Affiliations:** 1 Department of Public and Occupational Health, Amsterdam Public Health Research Institute, Amsterdam UMC, Amsterdam, The Netherlands; 2 Center for Nutrition, Prevention and Health Services, National Institute for Public Health and the Environment, P.O. Box 1, 3720 BA Bilthoven, The Netherlands; 3 Department of Public Health, Erasmus University Medical Center Rotterdam, Rotterdam, The Netherlands

**Keywords:** Occupational physical activity, Leisure-time physical activity, Overweight, Obesity, Self-rated health, Interaction, Workers’ health surveillance

## Abstract

**Purpose:**

To investigate the combined effects of occupational physical activity (OPA) and either overweight/obesity or low levels of leisure-time vigorous physical activity (LTVPA) on self-rated health.

**Methods:**

A longitudinal study was performed among 29,987 construction workers with complete data on 2 Workers’ Health Surveillance Programs during 2010–2018. Self-reported OPA involved strenuous work postures and manual material handling. Low level of LTVPA was defined as self-reported vigorous activity for less than three times per week lasting at least 20 min per session. Overweight and obesity were based on Body Mass Index (BMI) (25.0 ≤ BMI < 30.0 kg/m^2^ and BMI ≥ 30.0 kg/m^2^, respectively) using measured body height and weight. Self-rated health was measured using a single item question. Logistic regression analysis was used to investigate the associations between the separate risk factors at baseline and self-rated health at follow-up. The combined effects of demanding OPA and either overweight/obesity or low level of LTVPA on self-rated health were analyzed using the relative excess risk due to interaction (RERI).

**Results:**

Mean follow-up duration was 31.7 (SD = 14.9) months. Construction workers with strenuous work postures (OR 1.35 95% CI 1.25–1.46), manual material handling (OR 1.29 95% CI 1.19–1.40), obesity (OR 1.31 95% CI 1.17–1.47) and low LTVPA (OR 1.13 95% CI 1.01–1.25) were more likely to report poor self-rated health at follow-up. No statistically significant interaction effects were found for OPA and obesity or low LTVPA.

**Conclusions:**

OPA, obesity and low level of LTVPA were separate risk factors for poor self-rated health, but did not appear to have a synergistic effect.

## Introduction

Workers with a low socioeconomic position (SEP), many of whom are blue-collar workers, have higher mortality rates and a poorer health compared to workers with a high SEP (Dieker et al. 2019; Moor et al. 2017). Several work-related determinants and individual lifestyle behaviors play a role in the relatively poor health of low SEP workers. As to the work-related determinants, blue-collar workers more often work in disadvantaged work environments, including demanding occupational physical activities (van der Beek and Kunst 2019). For example, construction workers, who are typically blue-collar workers, are exposed to occupational physical activities (OPA) (Azevedo et al. 2020) with manual materials handling, bending and twisting of the trunk and exposure to whole-body vibration, making them at increased risk for poor (musculoskeletal) health (Badarin et al. 2021; Burstrom et al. 2015; Coenen et al. 2014; da Costa and Vieira 2010; Hiesinger and Tophoven 2019; Merkus et al. 2019). Indeed, in contrast to the favorable health effects of leisure-time physical activity (LTPA), high levels of OPA have been shown to impair health (Coenen et al. 2018; Gupta et al. 2020; Holtermann et al. 2012). These opposite health effects of physical activity in the different domains of leisure time versus those of physical activity at work is termed as the physical activity paradox (Holtermann et al. 2012).

In addition to the explanation of the contribution of demanding OPA to the poor health among blue-collar workers, a higher prevalence of leisure-time physical inactivity among blue-collar workers, including construction workers, is seen compared to generally higher educated, white-collar workers (Boal et al. 2020; Gilson et al. 2019). This lower level of LTPA may be partly caused by exhaustion due to demanding OPA of workers in manual jobs, leaving little energy for performing other physical activities outside working hours. Also, blue-collar workers may believe that they already meet the recommended level of physical activity while at work and thus lack motivation to also engage in LTPA (Brownson et al. 2001; Rasmussen et al. 2018). In addition, linked to leisure-time physical inactivity, blue-collar workers including the specific group of construction workers, have higher rates of overweight or obesity compared to white-collar occupational groups (Alavinia et al. 2009; Boal et al. 2020; Hanson et al. 2021; Harris et al. 2011; Myers et al. 2021; Willett et al. 2013).

Considering the adverse health effects of overweight and low levels of leisure-time physical activity (Cecchini et al. 2010; Kaleta et al. 2006, 2009; Kraja et al. 2013; Molarius et al. 2007; Rongen et al. 2014; Shields and Shooshtari 2001; Viester et al. 2013; WHO 2004), effective promotion of LTPA and a healthy body weight as well as initiatives to target the reduction of demanding OPA among blue-collar workers can contribute to improvement of their health (Korshoj et al. 2016; Viester et al. 2018; Volandis 2018). Besides, the proven separate health effects of OPA on one hand and LTPA and body mass index (BMI) on the other hand, these factors may interact with each other. To explain, workers with demanding OPA can be more vulnerable for negative health effects of overweight and low levels of leisure-time physical activity, implying a moderating role of these factors. This was confirmed in a study among construction workers where overweight was found to be moderately associated with an increased prevalence of musculoskeletal symptoms, modified by OPA (Viester et al. 2013). A possible explanation might be that demanding OPA and a high BMI both increase mechanical forces and moments on the joints, which are known risk factors for MSDs, and could thus in combination have increased the adverse effects on health (Pietiläinen et al. 2011; Thijssen et al. 2015). Also, OPA are mostly performed during the entire working hours, potentially with insufficient time to recover, and thus may not lead to the cardiorespiratory health benefits (Howley 2001; Savinainen et al. 2004) as is valid for leisure-time vigorous physical activity (LTVPA) (Costigan et al. 2015), which may add to the cardiovascular health risk of low levels of leisure-time physical activity and overweight or obesity.

In sum, the underlying mechanisms indicate that the combination of OPA and overweight/obesity or low level of LTVPA may have a stronger effect on self-rated health than the sum of each of the these separate factors. Until now, only a small number of studies have been conducted on the combined effects of these factors on different health outcomes (Hoven and Siegrist 2013; Moor et al. 2017; Robroek et al. 2017; Tonnon et al. 2019). To gain more insight into the role of LTVPA and overweight/obesity in the relation between high OPA and self-perceived health, this study aimed to investigate the combined effects of a high OPA and either overweight/obesity or low level of LTVPA on self-rated health.

## Methods

The dataset used in this longitudinal study consisted of Dutch construction workers who participated in two Workers’ Health Surveillance Programs (WHS) conducted by Occupational Health Services (OHS) throughout the Netherlands in the period between 2010 and 2018. All construction workers were invited to a WHS every 4 years, those aged 40 years or older were invited every 2 years, and those with high physically demanding jobs (e.g. scaffolders) were invited every year (Volandis 2017). The WHS consisted of a physical examination and a questionnaire. The questionnaire was sent to the home address of the worker together with a call for the physical examination at the occupational health service. The worker was asked to fill in the questionnaire and take the completed questionnaire to the occupational health service location where the physical examination was performed, including the measurement of body weight and height. By an explicit statement included in the questionnaire, respondents consented to the use of their data for scientific purposes. Data from the WHS over the period 2010 until 2018 were used, since questions about lifestyle and self-rated health were introduced from 2010 onwards (Arbouw 2010). For this study, male construction site workers were included, and those with two WHS measurements. Exclusion criteria were: workers with only administrative tasks and workers with only one WHS measurement.

### Variables

#### Occupational physical activity

Four questions, measured at baseline (i.e. the first WHS), to be answered on a dichotomous scale (yes/no) were used to assess OPA (Tonnon et al. 2019). Two questions referred to strenuous work postures, the other two involved manual material handling. Strenuous work postures included the questions ‘During your work, do you often have to work for a prolonged period of time in a kneeling or crouching position?’ and ‘Do you often have to work for a prolonged period of time in an uncomfortable position?’. Material handling was measured using the questions: ‘During your work, do you often have to lift, push, pull or carry heavy loads?’ and ‘During your work, do you often have to exert great force?’ (Tonnon et al. 2019). If one or more of the two questions was answered positively, then the respondent was considered as being exposed to strenuous work postures or manual material handling, respectively. The respondents who answered both questions per physical workload construct with ‘no’ were considered to have no strenuous work postures or manual material handling and were used as reference category.

#### Overweight

Body mass index (BMI) was calculated using measured body height and body weight during the physical examination at baseline. Using the WHO guidelines, BMI was categorized in healthy weight (18.5 ≤ BMI < 25.0 kg/m^2^), overweight (25.0 ≤ BMI < 30.0 kg/m^2^), and obesity (BMI ≥ 30.0 kg/m^2^) (WHO 2004). As less than 1% of the population had underweight, we excluded these participants. Healthy weight was used as reference category.

#### Leisure-time vigorous physical activity

In this study, LTVPA was measured at baseline by means of a single question to the frequency per week of LTVPA in the past month: ‘How many days during the past month were you involved in vigorous physical activity (sports) that caused sweating and lasted at least 20 min?’. Respondents were considered to have low level of LTVPA if they were vigorously active for less than three times per week. Respondents who reported being vigorously active for three or more times per week were considered to have high level of LTVPA and were used as reference category. This cut-off point was based on an earlier Dutch recommendation (Kemper et al. 2000) to perform at least three times per week vigorous intensity activities lasting at least 20 min per session to maintain or improve physical fitness. As the question on LTVPA in the WHS was based on this former guideline, we also used this for the current study. Based on the recent WHO guideline (WHO 2020) one could argue that the minimum of 60 min is insufficient to achieve fitness and health benefits. However, as the recent WHO guideline does not explicitly distinguish between domains and thereby offers room for physical activity at work, which is common among the construction workers, we decided for the cut-off point based on the earlier LTVPA recommendation.

#### Self-rated health

Self-rated health was measured using a single question (Arbouw 2010; Jylha 2009), both at baseline and at follow-up (T1): ‘Do you feel healthy?’. If the respondent reported ‘yes’, the respondent was considered having a good self-rated health. If the respondent answered ‘no’, the respondent was considered having poor self-rated health. Self-rated health at follow-up was used as the dependent variable in this study.

#### Covariates

Several sociodemographic, behavioral and workload factors were included as covariates based on previous associations found in the literature. Age and working hours were taken into account as covariates as was done earlier (Kaikkonen et al. 2009; Proper et al. 2020). Because of a lot of missing data and similar (low) educational level of the included construction workers (Proper et al. 2020), education was not used as covariate. Based on the job-demand-control-support (Johnson and Hall 1988) and the effort-reward model (Siegrist 1996), a number of psychosocial workload factors were considered as covariates, namely job control, job demands, social support, rewards, job satisfaction, and work insecurity. Job control was measured by means of two questions (Can you decide yourself, how you do your job?, Can you influence the work speed?); work demands was also measured by two questions (Do you often work under time pressure, Do you have a lot of work to do?). Participants were considered having a low job control or high job demands if they answered at least one of the two questions with ‘yes’. Social support was measured by means of three questions (supported by supervisor, sphere at work, sufficient time for consultation) and was considered as low if a respondent answered ‘yes’ to at least one of the three questions. Rewards was measured by two questions (feeling appreciated, sufficient reward) and considered low if at least one question was answered with ‘no’. Job satisfaction and work insecurity were measured using a single question: “In general do you feel satisfied in your current job?” (yes/no) and “Does this job/employer offer you sufficient work security?”. All questions to psychosocial workload factors used dichotomous answer categories (yes/no). Due to the number of available psychosocial workload factors, they were only included in the models if the regression coefficient changed more than 10%. Lifestyle factors other than LTPA and BMI were considered as potential covariates, i.e. smoking (never, former, or current smoker) and alcohol consumption (0, 1–7, or > 7 glasses per week) (Gezondheidsraad 2015).

### Statistical analysis

**Fig. 1:**
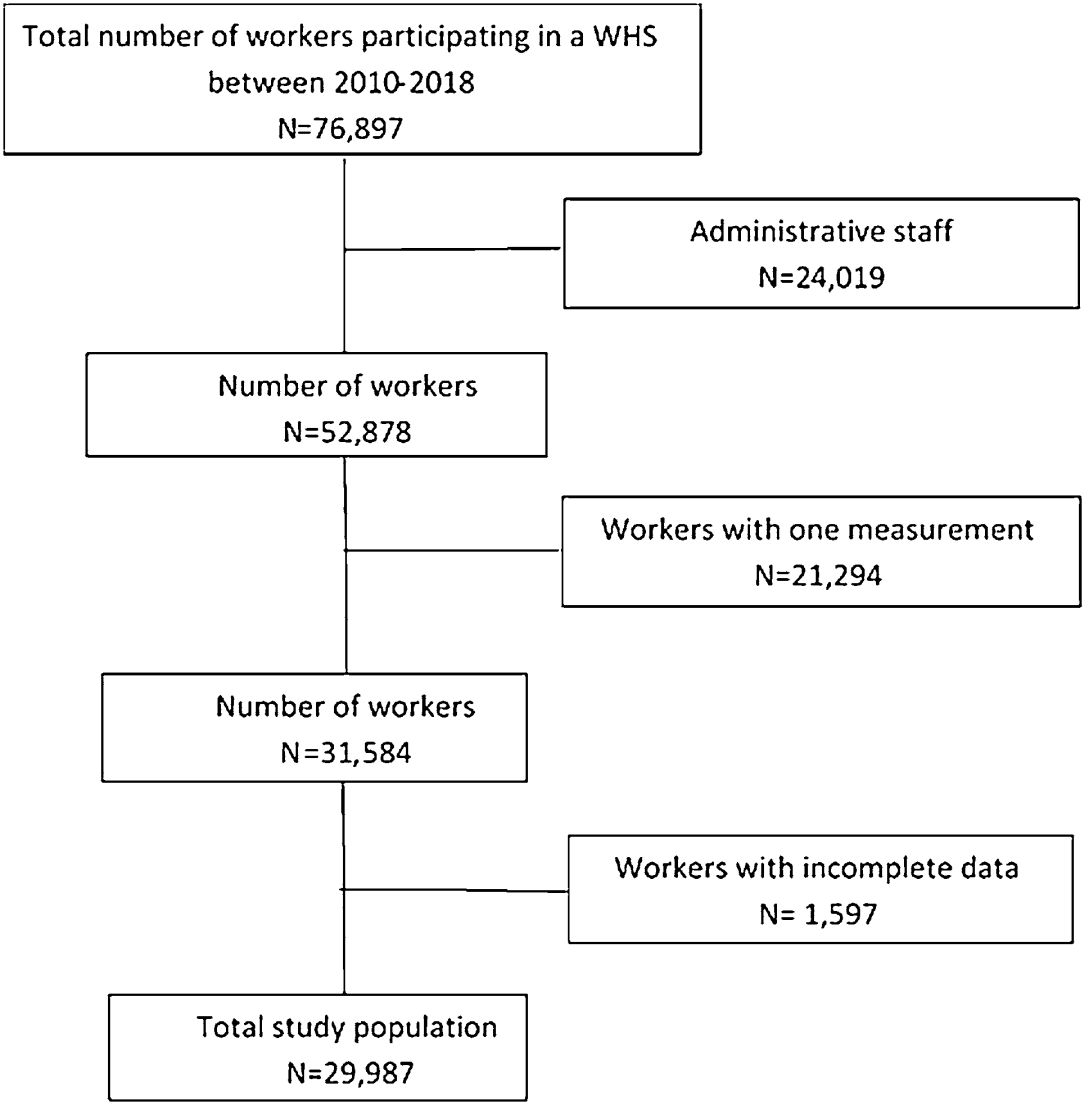
Flowchart of the study population

In total, 76,897 workers participated in a WHS between 2010 and 2018. Administrative workers and workers with missing values on job function were excluded (*n* = 24,019), followed by the exclusion of workers with single measurements (*n* = 21,294). For the purpose of this study, only the two most recent WHS measurements of each worker were used, thus previous measurements were excluded. In total, 31,584 workers with two WHS measurements were included (Fig. 1). Incomplete data on self-rated health (*n* = 115), physical workload (*n* = 302), BMI (*n* = 125), physical activity (*n* = 196), and covariates (*n* = 1401) were excluded. This resulted in exclusion of 1597 workers due to missing data on 1 or more variables. In total, 29,987 construction workers with complete data on 2 WHS measurements were included for analyses in this study. Baseline characteristics of the total study population were presented by descriptives and stratified according to self-rated health at follow-up (T1). Logistic regression analyses were used to investigate the associations between demanding OPA, overweight/obesity and low levels of LTVPA at baseline and poor self-rated health at follow-up. Different models were used to adjust for variables, which were all measured at baseline. Model 1 was adjusted for age, time to follow-up, working hours and lifestyle factors (BMI, physical activity, smoking and alcohol consumption). Model 2 was additionally adjusted for psychosocial workload factors (demands, support, rewards, and job security). Finally, self-rated health at baseline was also added in model 3.

To analyze the additive interaction effects of overweight/obesity and low level of LTVPA in the association of OPA at baseline and self-rated health at follow-up, variables were created including the possible four combinations of OPA constructs on one hand and overweight/obesity and levels of LTVPA on the other. For example, the following categorical variable was created for the combination of LTVPA and strenuous work postures: high level of LTVPA and no strenuous work postures; high level of LTVPA and strenuous work postures; low level of LTVPA and no strenuous work postures; low level of LTVPA and strenuous work postures. Logistic regression analyses were conducted to investigate the associations between the combined variables and self-rated health at follow-up. The odds ratios (OR) of the combined associations were used to calculate the relative excess risk due to interaction (RERI) and the corresponding 95% confidence interval (CI), using this formula for each lifestyle factor:

RERI overweight and strenuous work postures = OR(overweight + high strenuous work postures) − OR(overweight + low strenuous work postures) − OR(normal weight + high strenuous work postures) + 1.

The same formula was used to calculate the RERI for low levels of LTVPA with strenuous work postures. Similar combinations were made for overweight/obesity and low levels of LTVPA with the OPA variable manual material handling. These RERI analyses were fully adjusted for all of the variables described earlier. Statistical analyses were carried out in IBM SPSS Statistics version 26.

## Results

The median age of the construction workers was 49.0 (IQR = 14) years at baseline (Table 1), and median follow-up duration was 26.0 (IQR = 19 min–max = 1–107) months. In total, 50.6% had overweight and 17.3% obesity, and 83% was considered insufficiently vigorously active at baseline. Strenuous work postures were reported by 41.4% of the participants, and 54.8% was exposed to manual material handling. Of all participants, 11.3% reported poor self-rated health at follow-up.

**Table 1:** Baseline characteristics of 29,987 construction workers stratified by self-rated health at follow-up

		Total *N* = 29,987	Good self-rated health (T1) *N* = 26,590)	Poor self-rated health (T1) *N* = 3397
Age (years)	Median (IQR)	49.0 (14)	49.0 (14)	52.0 (11)
Working hours	Mean (SD)	40.0 (0)	40.0 (0)	40.0 (0)
Follow-up duration (months)	Median (IQR)^a^	26.0 (19)	26.0 (20)	25.0 (13)
OPA^b^ Strenuous work postures (%) Material handling (%)	*N* (%)	12,426 (41.4) 16,446 (54.8)	10,627 (40.0) 14,300 (53.8)	1799 (53.0) 2146 (63.2)
Poor self-rated health	*N* (%)	2788 (9.3)	1607 (6.0)	1181 (34.7)
BMI^c^ (kg/m^2^)	Median (IQR)	26.5 (4.5)	26.5 (4.5)	26.9 (4.8)
BMI	*N* (%)			
Healthy Overweight Obesity		9638 (32.1) 15,170 (50.6) 5179 (17.3)	8697 (32.7) 13,449 (50.6) 4444 (16.7)	941 (27.7) 1721 (50.7) 735 (21.6)
Low level of LTVPA^d^	*N* (%)	24,904 (83.0)	21,992 (82.7)	2912 (85.7)
Smoking	*N* (%)			
Never Former Yes		11,454 (38.2) 10,098 (33.7) 8435 (28.1)	10,352 (38.9) 8820 (33.2) 7418 (27.9)	1102 (32.4) 1278 (37.6) 1017 (29.9)
Alcohol consumption	*N* (%)			
0 glasses per week 1–7 glasses per week > 7 glasses per week		4656 (15.5) 12,800 (42.7) 12,531 (41.8)	4010 (15.1) 11,386 (42.8) 11,194 (42.1)	646 (19.0) 1414 (41.6) 1337 (39.4)
Work related factors	*N* (%)			
Low job control High job demands Low social support High job insecurity Low rewards Low job satisfaction		9320 (31.1) 16,700 (55.7) 4501 (15.0) 6998 (23.3) 12,500 (41.7) 1115 (3.7)	8084 (30.4) 14,517 (54.6) 3640 (13.7) 5863 (22.0) 10,699 (40.2) 788 (3.0)	1236 (36.4) 2183 (64.3) 861 (25.3) 1135 (33.4) 1801 (53.0) 327 (9.6)

*SD* standard deviation

^a^Minimum and maximum follow-up duration of the total population is 1–107 months, for those with good SRH 1–107 months and for those with poor SRH 1–104 months

^b^OPA: occupational physical activity, here defined by strenuous work postures and material handling

^c^BMI: body mass index; overweight: BMI 25–29.9 kg/m^2^, obesity = BMI ≥ 30 kg/m^2^

^d^Low level of LTVPA (leisure time vigorous physical activity): < 3 days per week for at least 20 min per session

Table 2 shows that construction workers with high strenuous work postures (OR 1.35, 95% CI 1.25–1.46) or manual material handling (OR 1.29, 95% CI 1.19–1.40) at baseline were more likely to report poor self-rated health at follow-up than those without demanding OPA, even after adjustment for covariates and self-rated health at baseline. Overweight at baseline was significantly associated with poor self-rated health at follow-up in the univariate model, but not after adjustment for covariates (see Table 2). For obesity, significant associations with poor self-rated health at follow-up were found in all models, including the fully adjusted model (OR 1.31, 95% CI 1.17–1.47). Construction workers with low levels of LTVPA were significantly more likely to report poor self-rated health at follow-up (OR 1.13, 95% CI 1.01–1.25) in all models.

**Table 2:** Associations of OPA, overweight/obesity and low level of LTVPA at baseline with poor self-rated health at follow-up among 29,987 construction workers

	Poor self-rated health (*n* = 3397/29,987)			
	Univariate model OR (95% CI)	Adjusted model 1^a^ OR (95% CI)	Adjusted model 2^b^ OR (95% CI)	Adjusted model 3^c^ OR (95% CI)
OPA				
Strenuous work postures Manual material handling	1.69 (1.57–1.81) 1.47 (1.37–1.58)	1.70 (1.58–1.83) 1.58 (1.47–1.71)	1.46 (1.35–1.57) 1.34 (1.24–1.44)	1.35 (1.25–1.46) 1.29 (1.19–1.40)
BMI				
Overweight Obesity	1.18 (1.09–1.29) 1.53 (1.38–1.69)	1.08 (1.00–1.18) 1.38 (1.24–1.54)	1.08 (0.99 -1.18) 1.39 (1.25–1.54)	1.07 (0.98–1.17) 1.31 (1.17–1.47)
Low level of LTVPA	1.25 (1.13–1.39)	1.17 (1.05–1.29)	1.23 (1.11–1.37)	1.13 (1.01–1.25)

Low level of LTVPA (leisure time vigorous physical activity): < 3 days per week for at least 20 min per session

*OR* Odds ratio, *CI* confidence interval, *OPA* occupational physical activity, *BMI* body mass index; overweight: BMI 25–29.9 kg/m^2^, obesity = BMI ≥ 30 kg/m^2^

^a^Adjusted for age, time to follow-up, working hours and lifestyle factors BMI, physical activity, smoking and alcohol consumption

^b^Additionally adjusted for workload factors namely job demands, psychosocial demands, support, rewards and job security

^c^Additionally adjusted for self-rated health at baseline

Table 3 shows the results of the interaction effects of both BMI categories and low levels of LTVPA with OPA on poor self-rated health. It appeared that none of the RERIs showed a statistically significantly additive interaction effect, neither for the combination of high manual material handling or strenuous work postures and overweight/obesity on poor self-rated health, nor for the combination of high manual material handling or strenuous work postures and low levels of LTVPA on poor self-rated health.

**Table 3:** Interaction effects of overweight/obesity and low levels of LTVPA with high OPA on poor self-rated health among 29,987 construction workers

	Poor self-rated health		
	*n*	OR (95% CI)	RERI (95% CI)
BMI and strenuous work postures			
Healthy body weight and no strenuous work postures Healthy body weight and strenuous work postures	5527 4111	1.00 1.44 (1.24–1.66)	
Overweight and no strenuous work postures Overweight and strenuous work postures	8893 6277	1.14 (1.00–1.29) 1.46 (1.28–1.66)	− 0.12 (− 0.35 to 0.11)
Obesity and no strenuous work postures Obesity and strenuous work postures	3141 2038	1.32 (1.13–1.55) 1.89 (1.61–2.23)	0.13 (− 0.19 to 0.46)
LTVPA and strenuous work postures			
High level of LTVPA and no strenuous work postures High level of LTVPA and strenuous work postures	2668 2415	1.00 1.27 (1.04–1.55)	
Low level of LTVPA and no strenuous work postures Low level of LTVPA and strenuous work postures	14,893 10,011	1.08 (0.92–1.27) 1.48 (1.26–1.74)	0.13 (− 0.11 to 0.36)
BMI and manual material handling			
Healthy body weight and no material handling Healthy body weight and material handling	4151 5487	1.00 1.27 (1.10–1.48)	
Overweight and no material handling Overweight and material handling	6925 8245	1.09 (0.94–1.26) 1.34 (1.17–1.54)	− 0.01 (− 0.22 to 0.20)
Obesity and no material handling Obesity and material handling	2465 2714	1.20 (1.00–1.44) 1.76 (1.50–2.07)	0.29 (0.00–0.57)
LTPA and manual material handling			
High level of LTVPA and no material handling High level of LTVPA and material handling	1894 3189	1.00 1.38 (1.11–1.70)	
Low level of LTVPA and no material handling Low level of LTVPA and material handling	11,647 13,257	1.18 (0.98–1.43) 1.51 (1.25–1.83)	− 0.05 (− 0.32 to 0.23)

Low level of LTVPA (leisure time vigorous physical activity): < 3 days per week for at least 20 min per session

*OR* Odds ratio, *CI* confidence interval, *RERI* relative excess risk due to interaction, *OPA* occupational physical activity, *BMI* body mass index; healthy body weight: BMI 18.5–24.9 kg/m^2^, overweight: BMI 25–29.9 kg/m^2^, obesity = BMI ≥ 30 kg/m^2^

## Discussion

Construction workers with obesity were at increased risk for future poor self-rated health compared to their colleagues with a healthy body weight, as were those with low levels of LTVPA compared to their colleagues with high levels of LTVPA. Also, workers with demanding OPA were more likely to rate their health as poor at follow-up. However, the combination of demanding OPA with either overweight/obesity or low level of LTVPA did not yield a statistically significant interaction, implying that the combined effects of demanding OPA and either overweight/obesity or low levels of LTVPA were not stronger than the sum of the separate effects.

Our findings that demanding OPA, obesity and low levels of LTVPA were significantly associated with poor self-rated health are in line with previous studies (de Breij et al. 2020; Proper et al. 2020; van Oostrom et al. 2021). Using the same dataset of construction workers, Proper et al. also found significant associations for high OPA and unhealthy behaviors with poor self-rated health (Proper et al. 2020). Another recent study, among lower educated workers from various economic sectors, also confirmed that those with obesity or being physically inactive in leisure time were more likely to report poor health (van Oostrom et al. 2021). In addition, in a study to identify work characteristics associated with health outcomes in older workers, it was found that exposure to high OPA was significantly associated with poorer self-rated health, especially in lower educated workers (de Breij et al. 2020). Thus, based on our and earlier research, there seems to be no debate about the adverse effects of OPA, obesity and low levels of LTVPA on poor self-rated health.

The scientific discussion is rather about the interplay between work-related and lifestyle-related factors in the relation with workers’ health. Our hypothesis for an added effect was partly based upon the physical activity health paradox (Coenen et al. 2018; Gupta et al. 2020; Holtermann et al. 2012) with opposite health effects of physical activity at work versus in leisure time. It was assumed that when a worker is exposed to both risk factors, i.e. demanding OPA and low levels of LTVPA, the adverse effects may add. This is especially the case with construction workers who because of their work have to deal with high levels of OPA, and as a result may feel too tired to perform any kind of aerobic physical activity in leisure time. However, in the current study, we could not confirm such synergistic effect of physical activity in the two domains. For overweight/obesity and OPA, which share a common mechanical pathway in the development of musculoskeletal disorders (Robroek et al. 2017; Runhaar et al. 2011), we did not observe a synergistic effect. This may suggest that the construction workers with demanding OPA are not more susceptible to the negative effects of low levels of LTVPA and overweight/obesity. In the explanation for the lack of an interaction, the intensity of LTPA may play a role. As the measure involved vigorous physical activity and workers were considered to have low levels of LTVPA if they performed less than 3 times per week 20 min vigorous physical activity, they still could be physically active at a moderate intensity and sufficient frequency, thereby achieving health benefits (Bull et al. 2020; WHO 2020). Because we, unfortunately, did not have such detailed data on the intensity, duration and frequency of physical activity in leisure time, but this may play an explanatory role. Further research into the interaction between occupational and leisure-time physical activity on self-rated health is thus needed. Still, it must be taken into consideration that the effect of OPA and LTPA cannot be disentangled to the full extent (Hulshof et al. 2021a; Hulshof et al. 2021b). Also, the use of a general measure of health in our study may explain the lack of the hypothesized added effect of demanding OPA and LTVPA or overweight/obesity. In contrast with our findings, two previous studies showed a synergistic effect of a high physical workload and obesity on future work ability and work disability benefits due to musculoskeletal disorders (Robroek et al. 2017; Tonnon et al. 2019). These two studies were also performed in construction workers, but indeed used another outcome, which may explain the contrasting results with the current study. In addition, differences in confounders adjusted for may explain the contrasting findings between our and earlier studies. In our study, we adjusted for additional covariates, including time to follow-up, working hours, and BMI and physical activity. From the results in Table 2, it indeed appeared that, overall, each successive model with additional confounders resulted in a smaller effect size. Furthermore, Table 3 confirms that the odds ratios for the exposure of two risk factors were consistently higher than the odds ratios in case of a single exposure, but the odds ratio found for the combined exposure was lower for poor self-rated health than previously reported for poor work ability (Tonnon et al. 2019). Despite the large sample size, the statistical power for finding a significant effect of a combination of the two risk factors was also somewhat lower. On the assumption that a general measure of health can include various health aspects and diseases, it is plausible that this has led to weakening of the effect. To gain better understanding of the separate and combined effects of physical workload and either overweight/obesity or insufficient vigorous physical activity on the interpretation of self-rated health, more research is needed.

### Strengths and limitations

This study is distinctive as it is one of the few on the combined effects of lifestyle factors and physical workload on self-rated health. The strengths of this study are its longitudinal design, large sample size, objective measurement of BMI, and the multiple variables adjusted for. These strengths enabled us to investigate the combined effects of high OPA and either overweight/obesity or low level of LTVPA on future self-rated health in a large group of construction workers.

Although body height and weight were measured objectively during the physical examination, BMI may not be the preferred anthropometric indicator of all health outcomes, including undiagnosed diabetes type 2 (Xu et al. 2013). Especially in muscular persons, such as construction workers with high physically demanding work, more workers may be misclassified as obese (Schneider et al. 2010) and the effect of obesity on self-rated health in the current study may, therefore, have been underestimated. Still, BMI is a widely used and acceptable indicator of obesity at the population level (Scafoglieri et al. 2014), which was also confirmed in a study by Gutin (Gutin 2018).

Despite the large dataset with a wide set of variables, most variables, including the main determinants (OPA and LTVPA) and the outcome measure (self-rated health), were based on self-report, which may be imprecise due to differences in interpretation (Prince et al. 2008; Troiano et al. 2008). For example, LTPA is mostly overestimated (Lechner et al. 2006) and it is plausible that especially construction workers, whose work require certain levels of activity, may overestimate their actual level of physical activity. However, as we used vigorous activity as indicator for LTPA and considering that 83% was classified as having low levels of LTVPA, bias due to overreporting is likely to be small. Added to this, the question about vigorous activity included the example of performing sports, but did not make explicit distinction between leisure-time or occupational physical activity. As physical activity in these two domains have shown potentially opposite health effects (Holtermann et al. 2018), the use of a non-explicit domain related physical activity question could have led to a weakening of the effect on self-rated health. To reduce this potential weakening effect, we decided to use the question that asks to vigorous intensity physical activity rather than to moderate intensity physical activity, as we assumed one can more easily recall vigorous activities, such as sports, than activities at a lower intensity that are more integrated in daily functioning. Based on a review, higher correlations with objectively measured activity data were reported for vigorous compared to moderate physical activity (van Poppel et al. 2010). Still, it is recommended to use more valid measurement instruments that distinguish different domains of physical activity. Despite the fact that the single item question to self-rated health has been used since decades (Streib et al. 1958) and also increasingly in epidemiological research (Jylha 2009; Rohrer et al. 2005), the use of a dichotomous scale rather than categorical answer options ranging from poor to very good, may have led to a higher proportion of workers rating their health as good. The relatively low prevalence of 11% of self-rated poor health in our study population compared to 10–34% in the general Dutch adult population (CBS) can be an expression of this. However, this may also be explained by a healthy worker effect in our study population of construction workers, since they all were able to work. On the other hand, despite the use of three to five answer categories, which has more nuance than a dichotomy, these categories are often collapsed into a dichotomous variable of good versus less than good or poor health (Manor et al. 2000). In a study comparing the dichotomous self-rated variable as outcome compared to categorical variables, results appeared to be similar suggesting the risk for bias due to imprecision small (Manor et al. 2000). Although we used a longitudinal study design with self-rated health measured at a later moment in time than the risk factors, causality cannot be guaranteed. To increase the plausibility of causal relationships, a study is needed among workers with a good self-reported health at baseline, where different trajectories of (combined) risk factors over time are studied in their association with poor self-rated health at follow-up. However, as the risk factors were measured on average about 2.5 years before the health outcome, the likelihood for reversed causality is small. In this context, the average follow-up time of 32 months also raises the question of how much baseline OPA or LTVPA can affect self-rated health after so much time. As separate effects were found for the risk factors, this seems realistic.

The current study may have dealt with selection bias due to a healthy worker effect (Boschman et al. 2012; Siebert et al. 2001). Compared to workers with health problems becoming unemployed or employed in less physically demanding jobs, the current study population may have consisted of a selection of relatively healthy workers, who are still fit for the job of construction work. Assuming that these workers are physically stronger, they are at lower risk for health problems and have a higher self-rated health than the total working population. Considering the relatively high age of the population under study (mean: 47.3 years), it may even make them more prone to healthy worker selection than younger populations. This could have led to an underestimation of the associations.

### Practical implications

Due to the ageing workforce and the increasing retirement age, it becomes more and more important to keep the work force healthy and prevent the health burden associated with high OPA and unhealthy lifestyle factors for the worker, employer and society (Troelstra et al. 2020; Virkkunen et al. 2006). To support this, the Netherlands Society of Occupational Health has developed a guidance document with combined attention for work-related risk factors and lifestyle factors in workers’ health surveillance programs (Sluiter et al. 2013). By taking into account that high OPA, obesity and low levels of LTVPA all increase the risk of future poor self-rated health, worksite health promotion interventions focusing on these factors can each have beneficial effects. However, the current results do not imply that prevention strategies should focus on specific groups with a combination of obesity or low levels of LTVPA and OPA. More research is recommended to evaluate combined effects of physical workload factors and lifestyle factors, preferably with more objective measurements during follow-up and in different occupational groups.

## Conclusions

Construction workers with demanding OPA as well as those with obesity and low levels of LTVPA were more likely to report poor self-rated health at follow-up. However, the work-related and lifestyle-related factors did not appear to have a synergistic effect. Our results indicate that worksite health promotion interventions aimed at reducing OPA and/or improving LTVPA and reducing obesity can have beneficial health effects. The limited generalizability of the study results to male construction workers should be taken into account. Therefore, further research in other working populations is recommended.

## Data Availability

The data that support the findings of this study are available from the PAGO database at Volandis, the knowledge centre for sustainable employability in the construction sector in The Netherlands. The PAGO database contains 25 years of occupational safety and health data on construction workers in The Netherlands. Restrictions apply to the availability of these data, which were used under license for this study. Data requests for research purposes can be send by email to info@Volandis.nl. More information on data, derived products and reports is available via http://www.volandis.nl.

## References

[CR1] Alavinia SM, Van Den Berg TI, Van Duivenbooden C, Elders LA, Burdorf A (2009). Impact of work-related factors, lifestyle, and work ability on sickness absence among Dutch construction workers. Scand J Work Environ Health.

[CR2] Arbouw (2010) Questionnaire WHS construction industry

[CR3] Azevedo LM, Chiavegato LD, Carvalho CRF, Braz JR, Nunes Cabral CM, Padula RS (2020). Are blue-collar workers more physically active than white-collar at work?. Arch Environ Occup Health.

[CR4] Badarin K, Hemmingsson T, Hillert L, Kjellberg K (2021). Physical workload and increased frequency of musculoskeletal pain: a cohort study of employed men and women with baseline occasional pain. Occup Environ Med.

[CR5] Boal WL, Li J, Dong XS, Sussell A (2020). Health risk behavior profile of construction workers, 32 states, 2013 to 2016. J Occup Environ Med.

[CR6] Boschman JS, van der Molen HF, Sluiter JK, Frings-Dresen MH (2012). Musculoskeletal disorders among construction workers: a one-year follow-up study. BMC Musculoskelet Disord.

[CR7] Brownson RC, Baker EA, Housemann RA, Brennan LK, Bacak SJ (2001). Environmental and policy determinants of physical activity in the United States. Am J Public Health.

[CR8] Bull FC (2020). World Health Organization 2020 guidelines on physical activity and sedentary behaviour. Br J Sports Med.

[CR9] Burstrom L, Nilsson T, Wahlstrom J (2015). Whole-body vibration and the risk of low back pain and sciatica: a systematic review and meta-analysis. Int Arch Occup Environ Health.

[CR10] Cecchini M, Sassi F, Lauer JA, Lee YY, Guajardo-Barron V, Chisholm D (2010). Tackling of unhealthy diets, physical inactivity, and obesity: health effects and cost-effectiveness. Lancet.

[CR11] Coenen P, Gouttebarge V, van der Burght AS, van Dieen JH, Frings-Dresen MH, van der Beek AJ, Burdorf A (2014). The effect of lifting during work on low back pain: a health impact assessment based on a meta-analysis. Occup Environ Med.

[CR12] Coenen P, Huysmans MA, Holtermann A, Krause N, van Mechelen W, Straker LM, van der Beek AJ (2018). Do highly physically active workers die early? A systematic review with meta-analysis of data from 193 696 participants. Br J Sports Med.

[CR13] Costigan SA, Eather N, Plotnikoff RC, Taaffe DR, Lubans DR (2015). High-intensity interval training for improving health-related fitness in adolescents: a systematic review and meta-analysis. Br J Sports Med.

[CR14] da Costa BR, Vieira ER (2010). Risk factors for work-related musculoskeletal disorders: a systematic review of recent longitudinal studies. Am J Ind Med.

[CR15] de Breij S, Huisman M, Deeg DJH (2020). Work characteristics and health in older workers: educational inequalities. PLoS ONE.

[CR16] Dieker AC, IJzelenberg W, Proper KI, Burdorf A, Ket J, van der Beek AJ, Hulsegge G (2019). The contribution of work and lifestyle factors to socioeconomic inequalities in self-rated health-a systematic review. Scand J Work Environ Health.

[CR17] Gezondheidsraad (2015). Dossier alcohol. Achtergronddocument bij de Richtlijnen goede voeding.

[CR18] Gilson ND, Hall C, Holtermann A, van der Beek AJ, Huysmans MA, Mathiassen SE, Straker L (2019). Sedentary and physical activity behavior in "Blue-Collar" workers: a systematic review of accelerometer studies. J Phys Act Health.

[CR19] Gupta N (2020). The physical activity paradox revisited: a prospective study on compositional accelerometer data and long-term sickness absence. Int J Behav Nutr Phys Act.

[CR20] Gutin I (2018). In BMI we trust: reframing the body mass index as a measure of health. Soc Theory Health.

[CR21] Hanson GC (2021). A comparison of safety, health, and well-being risk factors across five occupational samples. Front Public Health.

[CR22] Harris JR, Huang Y, Hannon PA, Williams B (2011). Low-socioeconomic status workers: their health risks and how to reach them. J Occup Environ Med.

[CR23] Hiesinger K, Tophoven S (2019). Job requirement level, work demands, and health: a prospective study among older workers. Int Arch Occup Environ Health.

[CR24] Holtermann A, Hansen JV, Burr H, Sogaard K, Sjogaard G (2012). The health paradox of occupational and leisure-time physical activity. Br J Sports Med.

[CR25] Holtermann A, Krause N, van der Beek AJ, Straker L (2018). The physical activity paradox: six reasons why occupational physical activity (OPA) does not confer the cardiovascular health benefits that leisure time physical activity does. Br J Sports Med.

[CR26] Hoven H, Siegrist J (2013). Work characteristics, socioeconomic position and health: a systematic review of mediation and moderation effects in prospective studies. Occup Environ Med.

[CR27] Howley ET (2001). Type of activity: resistance, aerobic and leisure versus occupational physical activity. Med Sci Sports Exerc.

[CR28] Hulshof CTJ (2021). The effect of occupational exposure to ergonomic risk factors on osteoarthritis of hip or knee and selected other musculoskeletal diseases: a systematic review and meta-analysis from the WHO/ILO Joint Estimates of the Work-related Burden of Disease and Injury. Environ Int.

[CR29] Hulshof CTJ (2021). The prevalence of occupational exposure to ergonomic risk factors: a systematic review and meta-analysis from the WHO/ILO Joint Estimates of the Work-related Burden of Disease and Injury. Environ Int.

[CR30] Johnson JV, Hall EM (1988). Job strain, work place social support, and cardiovascular disease: a cross-sectional study of a random sample of the Swedish working population. Am J Public Health.

[CR31] Jylha M (2009). What is self-rated health and why does it predict mortality? Towards a unified conceptual model. Soc Sci Med.

[CR32] Kaikkonen R, Rahkonen O, Lallukka T, Lahelma E (2009). Physical and psychosocial working conditions as explanations for occupational class inequalities in self-rated health. Eur J Public Health.

[CR33] Kaleta D, Makowiec-Dąbrowska T, Dziankowska-Zaborszczyk E, Jegier A (2006). Physical activity and self-perceived health status. Int J Occup Med Environ Health.

[CR34] Kaleta D, Polanska K, Dziankowska-Zaborszczyk E, Hanke W, Drygas W (2009). Factors influencing self-perception of health status. Cent Eur J Public Health.

[CR35] Kemper H, Ooijendijk W, Stiggelbout M (2000). Consensus over de Nederlandse norm voor gezond bewegen. Tijdschrift Voor Gezondheidswetenschappen.

[CR36] Korshoj M, Ravn MH, Holtermann A, Hansen AM, Krustrup P (2016). Aerobic exercise reduces biomarkers related to cardiovascular risk among cleaners: effects of a worksite intervention RCT. Int Arch Occup Environ Health.

[CR37] Kraja F, Këlliçi I, Çakërri L (2013). Determinants of self-perceived health status in population-based studies. Albanian Med J.

[CR38] Lechner L, Bolman C, Van Dijke M (2006). Factors related to misperception of physical activity in The Netherlands and implications for health promotion programmes. Health Promot Int.

[CR39] Manor O, Matthews S, Power C (2000). Dichotomous or categorical response? Analysing self-rated health and lifetime social class. Int J Epidemiol.

[CR40] Merkus SL, Lunde LK, Koch M, Waersted M, Knardahl S, Veiersted KB (2019). Physical capacity, occupational physical demands, and relative physical strain of older employees in construction and healthcare. Int Arch Occup Environ Health.

[CR41] Molarius A, Berglund K, Eriksson C, Lambe M, Nordström E, Eriksson HG, Feldman I (2007). Socioeconomic conditions, lifestyle factors, and self-rated health among men and women in Sweden. Eur J Public Health.

[CR42] Moor I, Spallek J, Richter M (2017). Explaining socioeconomic inequalities in self-rated health: a systematic review of the relative contribution of material, psychosocial and behavioural factors. J Epidemiol Community Health.

[CR43] Myers S, Govindarajulu U, Joseph MA, Landsbergis P (2021). Work characteristics, body mass index, and risk of obesity: the National Quality of Work Life Survey. Ann Work Expo Health.

[CR44] Pietiläinen O, Laaksonen M, Rahkonen O, Lahelma E (2011). Self-rated health as a predictor of disability retirement—the contribution of ill-health and working conditions. PLoS ONE.

[CR45] Prince SA, Adamo KB, Hamel ME, Hardt J, Connor Gorber S, Tremblay M (2008). A comparison of direct versus self-report measures for assessing physical activity in adults: a systematic review. Int J Behav Nutr Phys Act.

[CR46] Proper KI, Cillekens B, Twisk JW, Coenen P, Robroek SJ, van Oostrom SH (2020). The mediating effect of unhealthy behaviors and body mass index in the relation between high physical workload and self-rated poor health in male construction workers. J Occup Environ Med.

[CR47] Rasmussen CL, Palarea-Albaladejo J, Bauman A, Gupta N, Nabe-Nielsen K, Jorgensen MB, Holtermann A (2018). Does physically demanding work hinder a physically active lifestyle in low socioeconomic workers? A compositional data analysis based on accelerometer data. Int J Environ Res Public Health.

[CR48] Robroek SJ, Järvholm B, Van Der Beek AJ, Proper KI, Wahlström J, Burdorf A (2017). Influence of obesity and physical workload on disability benefits among construction workers followed up for 37 years. Occup Environ Med.

[CR49] Rohrer JE, Pierce JR, Blackburn C (2005). Lifestyle and mental health. Prev Med.

[CR50] Rongen A, Robroek SJ, Schaufeli W, Burdorf A (2014). The contribution of work engagement to self-perceived health, work ability, and sickness absence beyond health behaviors and work-related factors. J Occup Environ Med.

[CR51] Runhaar J, Koes BW, Clockaerts S, Bierma-Zeinstra SM (2011). A systematic review on changed biomechanics of lower extremities in obese individuals: a possible role in development of osteoarthritis. Obes Rev.

[CR52] Savinainen M, Nygard CH, Ilmarinen J (2004). A 16-year follow-up study of physical capacity in relation to perceived workload among ageing employees. Ergonomics.

[CR53] Scafoglieri A, Clarys JP, Cattrysse E, Bautmans I (2014). Use of anthropometry for the prediction of regional body tissue distribution in adults: benefits and limitations in clinical practice. Aging Dis.

[CR54] Schneider HJ (2010). The predictive value of different measures of obesity for incident cardiovascular events and mortality. J Clin Endocrinol Metab.

[CR55] Shields M, Shooshtari S (2001). Determinants of self-perceived health. Health Rep.

[CR56] Siebert U, Rothenbacher D, Daniel U, Brenner H (2001). Demonstration of the healthy worker survivor effect in a cohort of workers in the construction industry. Occup Environ Med.

[CR57] Siegrist J (1996). Adverse health effects of high-effort/low-reward conditions. J Occup Health Psychol.

[CR58] Sluiter J, Weel A, Hulshof C (2013). Leidraad preventief medisch onderzoek van werkenden.

[CR59] Streib G, Schuman E, Philips B (1958). An analysis of the validity of health questionnaires. Soc Forces.

[CR60] Thijssen E, van Caam A, van der Kraan PM (2015). Obesity and osteoarthritis, more than just wear and tear: pivotal roles for inflamed adipose tissue and dyslipidaemia in obesity-induced osteoarthritis. Rheumatology.

[CR61] Tonnon SC, Robroek SR, van der Beek AJ, Burdorf A, van der Ploeg HP, Caspers M, Proper KI (2019). Physical workload and obesity have a synergistic effect on work ability among construction workers. Int Arch Occup Environ Health.

[CR62] Troelstra SA, Coenen P, Boot CR, Harting J, Kunst AE, van der Beek AJ (2020). Smoking and sickness absence: a systematic review and meta-analysis. Scand J Work Environ Health.

[CR63] Troiano RP, Berrigan D, Dodd KW, Masse LC, Tilert T, McDowell M (2008). Physical activity in the United States measured by accelerometer. Med Sci Sports Exerc.

[CR64] van der Beek AJ, Kunst AE (2019). How can we break the vicious circle between poor health and exit from paid employment?. Scand J Work Environ Health.

[CR65] van Oostrom SH, Nachat A, Loef B, Proper KI (2021). The mediating role of unhealthy behaviors and body mass index in the relationship between high job strain and self-rated poor health among lower educated workers. Int Arch Occup Environ Health.

[CR66] van Poppel MN, Chinapaw MJ, Mokkink LB, van Mechelen W, Terwee CB (2010). Physical activity questionnaires for adults: a systematic review of measurement properties. Sports Med.

[CR67] Viester L, Verhagen EA, Hengel KMO, Koppes LL, van der Beek AJ, Bongers PM (2013). The relation between body mass index and musculoskeletal symptoms in the working population. BMC Musculoskelet Disord.

[CR68] Viester L, Verhagen E, Bongers PM, van der Beek AJ (2018). Effectiveness of a worksite intervention for male construction workers on dietary and physical activity behaviors, body mass index, and health outcomes: results of a randomized controlled trial. Am J Health Promot.

[CR69] Virkkunen H, Härmä M, Kauppinen T, Tenkanen L (2006). The triad of shift work, occupational noise, and physical workload and risk of coronary heart disease. Occup Environ Med.

[CR70] Volandis (2017) Industry atlas 2017. Volandis, Harderwijk

[CR71] Volandis (2018). Industry atlas 2018.

[CR72] WHO (2004) Obesity: preventing and managing the global epidemic. Report of a WHO Consultation. WHO, Geneva 11234459

[CR73] WHO (2020). WHO guidelines on physical activity and sedentary behaviour.

[CR74] Willett WC, Hu FB, Thun M (2013). Overweight, obesity, and all-cause mortality. JAMA.

[CR75] Xu Z, Qi X, Dahl AK, Xu W (2013). Waist-to-height ratio is the best indicator for undiagnosed type 2 diabetes. Diabet Med.

